# *Clostridium butyricum* Reduces Obesity in a Butyrate-Independent Way

**DOI:** 10.3390/microorganisms11051292

**Published:** 2023-05-16

**Authors:** Jingyi Liao, Yaoliang Liu, Zhangming Pei, Hongchao Wang, Jinlin Zhu, Jianxin Zhao, Wenwei Lu, Wei Chen

**Affiliations:** 1 State Key Laboratory of Food Science and Technology, Jiangnan University, Wuxi 214122, China; 6200112047@stu.jiangnan.edu.cn (J.L.); 6200113197@stu.jiangnan.edu.cn (Y.L.); 7190112086@stu.jiangnan.edu.cn (Z.P.); hcwang@jiangnan.edu.cn (H.W.); wx_zjl@jiangnan.edu.cn (J.Z.); zhaojianxin@jiangnan.edu.cn (J.Z.); chenwei66@jiangnan.edu.cn (W.C.); 2 School of Food Science and Technology, Jiangnan University, Wuxi 214122, China; 3 National Engineering Research Center for Functional Food, Jiangnan University, Wuxi 214122, China

**Keywords:** *Clostridium butyricum*, microbial preparations, obesity, butyrate, purine, tryptophan, the gut microbiota

## Abstract

Accumulating evidence from recent studies links the gut microbiota to obesity, and microbiome therapy has been examined as a treatment. *Clostridium butyricum* (*C. butyricum*), an intestinal symbiont, protects the host from a range of diseases. Studies have shown a negative correlation between the relative abundance of *C. butyricum* and a predisposition for obesity. However, the physiological function and material basis of *C. butyricum* for obesity are unclear. Here, five *C. butyricum* isolates were administered to mice on a high-fat diet (HFD) to determine their anti-obesity effects. All isolates suppressed the formation and inflammation of subcutaneous fat, and the two effective strains considerably reduced weight gain and ameliorated dyslipidemia, hepatic steatosis, and inflammation. These positive effects were not achieved by increasing the concentration of intestinal butyrate, and the effective strains could not be replaced by sodium butyrate (NaB). We also discovered that oral supplementation with the two most effective strains changed the metabolism of tryptophan and purine and altered the composition of the gut microbiota. In summary, *C. butyricum* improved the metabolic phenotypes under the HFD by controlling the composition of the gut microbiota and modulating intestinal metabolites, thereby demonstrating its ability to fight obesity and providing a theoretical foundation for microbial preparations production.

## 1. Introduction

Obesity is a global epidemic characterized by an increase in central fat mass, chronic inflammation, and metabolic disorders. From 1975 to 2014, the global prevalence of obesity increased from 6.4% to 14.9% in women and from 3.2% to 10.8% in men [1]. Obesity is a leading cause of mortality, as it increases the risk of 21 diseases, including cardiovascular, gastrointestinal, respiratory, and neurological disorders [2]. As multifactorial diseases, obesity and its morbidities can be triggered by several factors. Long-term consumption of high-calorie foods without sufficient physical activity results in a positive energy balance and causes the onset of obesity [3]. Obesogenic factors also include stress and sleep deprivation, which also can disturb energy homeostasis by influencing appetite and energy metabolism [4,5]. 

As the study progressed, the link between gut symbionts and obesity has been increasingly elucidated, and the microbiota has become an intervention target for obesity treatments [5]. In 2006, Gordon et al., discovered a correlation between obesity and the relative abundances of *Bacteroidetes* and *Firmicutes* [6,7]. Compared to lean individuals, the obese have differences not only in the species diversity and composition of the gut microbiota, but also in the microbiota function. The capacity of gut microbes to obtain energy from food is greatly boosted in obese individuals [6]. In addition to affecting energy expenditure, the microbiota also alters the makeup of substances in the gut. Indole derivatives, bacterial metabolites of tryptophan, have also been linked to the etiology of obesity and associated metabolic consequences. These substances can act as endogenous aryl hydrocarbon receptor (AhR) agonists to suppress inflammation [8,9] and improve metabolism [10]. Moreover, short-chain fatty acids (SCFAs) generated by the bacterial fermentation of dietary fiber have been demonstrated to have anti-obesity benefits [11]. Butyrate is one of the main SCFAs in the intestine. It can act as an inhibitor of histone deacetylases or agonist of G-protein-coupled receptors to prevent diet-induced obesity and reduce energy intake by suppressing the appetite [12,13]. Studies have shown that the enteric concentration of butyrate of obese people has reduced, which may be related to the decrease of the abundance of butyrate-producing bacteria [14,15]. Therefore, supplementing butyrate-producing bacteria is a potential way to alleviate obesity.

*Clostridium butyricum* (*C. butyricum*) is one of butyrate-producing bacteria in the intestine, and it has the potential to alleviate a range of disorders such as inflammatory bowel disease (IBD), colitis, and obesity [16]. The abundance of *C. butyricum* can be decreased by high-fat diet (HFD), and it has been proved that oral administration of *C. butyricum* has the potential to alleviate obesity [17]. However, some studies have pointed out that butyrate-producing bacteria can help to lose weight without changing the level of butyrate in the intestine [18]. Therefore, whether certain *C. butyricum* strains can relieve the symptoms of obesity by producing butyric acid still needs further studies.

Based on the above background, the purpose of this study was to screen *C. butyricum* strains that can alleviate obesity and to explore their effects on intestinal substances such as SCFAs. To achieve this, five *C. butyricum* isolates were administered orally to mice with an HFD for 12 weeks, and sodium butyrate (NaB) was utilized to determine whether *C. butyricum* supplementation could be replaced. Body weight, serum lipid, adipose tissue weight, inflammation, and liver steatosis were utilized to evaluate the effects of the strains and NaB on ameliorating diet-induced obesity and the associated metabolic syndrome. To gain mechanistic insights into the anti-obesity action of *C. butyricum*, we also analyzed alterations in the gut microbiota and gut-derived compounds.

## 2. Materials and Methods

### 2.1. Preparation of C. butyricum Strains 

The five *C. butyricum* strains used in this experiment were preserved in the Jiangnan University Culture Collection of Food Microorganisms (Wuxi, China). *C. butyricum* FYNDL1T1 (L1T1) and FHBSJZ1T1 (Z1T1) were isolated from the feces of cow and dog, respectively, strains FHLJZD47T7 (47T7) and NXYCHL3M3 (L3M3) were isolated from healthy volunteers, and strain C20_1_1 (C20) was isolated from an obese volunteer. All strains were cultured anaerobically in a reinforced clostridial medium at 37 °C. The plate colony-counting method was used for the quantification of *C. butyricum*. In brief, the 1 mL bacterial suspension was diluted with PBS (added with 0.05% L- cysteine) in gradient, and then, 1 mL of diluted bacterial suspension was spread evenly on the solid culture medium. After 48 h of culture, the number of colonies was counted. Two liters of the bacterial suspension was concentrated according to the counting result. The specific operation was as follows: after the bacterial suspension was centrifuged at 8000 rpm for 20 min, the cultured bacterial cells were resuspended in PBS (added with 0.05% L-cysteine) at an approximate cell density of 5 × 10^8^ CFU/mL.

### 2.2. Animal Experiments

Sixty-four five-week-old male C57BL/6J mice (19–20 g; specific-pathogen-free (SPF)) were purchased from Charles River Laboratory Animal Technology Co., Ltd., (Jiaxing, China). These mice were divided into eight groups at random: normal chow (NC), HFD, NaB, and *C. butyricum*-treated groups (L1T1, Z1T1, C20, L3M3, and 47T7). Each of the above groups contained eight mice (*n* = 8). The mice were raised in an SPF-grade animal facility at 23 ± 2 °C and a 60% ± 20% humidity with a 12 h light–dark cycle. The intervention was initiated, after the mice were acclimated for 1 week. All the other groups were fed with an HFD, while the mice in the NC group were fed with a normal-chow diet (NCD) for 12 weeks. NCDs (total energy: 3.5 kcal/g; the fat provided 10% energy) and HFDs (total energy: 5 kcal/g; the fat provided 60% energy) were bought from TROPHIC Animal Feed High. During the 12-week intervention, each mouse in the NaB group was orally administered NaB (Sigma-Aldrich Trading Co., Ltd., Shanghai, China) once a day at 300 mg/kg, whereas the mice in the *C. butyricum*-treatment groups were orally received 0.2 mL bacterial suspension (a density of 5 × 10^8^ CFU/mL) once daily. As a control, all the mice in the NC and HFD groups were orally administered 0.2 mL of 0.9% saline. All experimental procedures were approved by the Animal Care and Use Committee of the Jiangnan University (JN. No20210430c0640915[100]).

### 2.3. Liver and Adipose Tissue Histology

Inguinal adipose tissue (iWAT; the subcutaneous adipose tissue was located at the junction of the lower part of the anterior abdominal wall and the leg) and liver tissues were embedded in paraffin after soaking in 4% paraformaldehyde for 24 h, and the paraffin blocks were sectioned and stained with hematoxylin and eosin (H&E). The stained sections were scanned using a digital slice scanner (Pannoramic MIDI II, 3DHISTECH, Budapest, Hungary) [19].

### 2.4. Detection of Biochemical Indicators

Serum biochemical indicators, including high-density lipoprotein cholesterol (HDL-C), low-density lipoprotein cholesterol (LDL-C), total cholesterol (TC), total triglyceride (TG), alanine aminotransferase (ALT), and aspartate aminotransferase (AST), were measured using a Beckman AU5800 automatic biochemical analyzer (Brea, CA, USA) [19].

### 2.5. Determination of Tissue Cytokines

The secretion of tumor necrosis factor (TNF)-α and interleukin (IL)-1β in the liver was determined by an enzyme-linked immunosorbent assay (ELISA), and the commercialized kits were from the R&D Systems (Minneapolis, MN, USA) [19].

### 2.6. Immunohistochemistry

Paraffin blocks embedded in subcutaneous adipose tissue were sectioned, dewaxed and hydrated. The hydrated sections were heated in an antigen-retrieval solution to recover antigens. After cooling to room temperature, the sections were washed with PBS. Endogenous peroxidase was subsequently quenched with a 3% H_2_O_2_ solution and blocked with 5% bovine serum albumin. An F4/80 primary antibody (Servicebio, Wuhan, China) was incubated overnight at 4 °C, and then, the unbound antibody was washed off with PBS. A secondary antibody (IgG H&L; Servicebio, Wuhan, China) was incubated at room temperature. After rinsing with DAB solution (Servicebio, Wuhan, China), the sections were counterstained with hematoxylin and dehydrated. The sections were photographed under a microscope and analyzed [20].

### 2.7. Detection of SCFAs

A mixture of cecal contents (50 mg) and 500 μL saturated NaCl solution were homogenized in a centrifuge tube, and acidified SCFAs (using 40 μL of 10% H_2_SO_4_) were then extracted with 1 mL ether. Subsequently, the ether layer containing SCFAs was separated by centrifugation (12,000 rpm, 15 min), and 0.25 g Na_2_SO_4_ was added to the supernatant to remove excess water. After centrifugation, the supernatant was used for subsequent analysis. Gas chromatography–mass spectrometry (GC-MS) (GCMS-QP2010 Ultra system, Shimadzu Corporation, Kyoto, Japan) equipped with an Rtx-Wax column was used to separate and detect three main SCFAs (propionic acid, acetic acid, and butyric acid) in the above samples. Helium was used as the mobile phase. The initial column temperature was 100 °C, which was increased to 140 °C at a heating rate of 7.5 °C per minute, then increased to 200 °C at a rate of 60 °C per minute and finally held at 200 °C for 3 min. SCFAs were detected in the full scan mode (scanning range of the mass-to-charge ratio: 33–110) [21].

### 2.8. Untargeted Metabolomics Analysis

Methanol, acetonitrile, and ultrapure water were mixed at a volume ratio of 2:2:1 as an extract solution, which was added into a centrifuge tube containing lyophilized mouse feces (50 mg). The mixture was homogenized and then centrifuged at 14,000 rpm for 20 min. The supernatant was collected for concentration (45 °C, 1.0 p, 4 h), and the concentrate was then reconstituted with 200 μL 80% methanol and centrifuged again. The filtered supernatant was used as the sample. The quality-control (QC) sample was prepared by mixing all samples in equal volumes, which was used to evaluate the stability of detection. The above samples were detected via ultra-performance liquid chromatography (LC) and using a Q-Exactive high-resolution mass spectrometer system (Thermo Fisher Scientific, Waltham, MA, USA) equipped with a C18 column (Waters UPLC BEH C18– 2.1 × 100 mm, 1.7 µm). In the positive ion detection mode, mobile-phase solutions A and B were 0.1% formic acid in water and acetonitrile, respectively. For the negative ion detection mode, solution A was a 5 mM ammonium acetate solution, and solution B was an acetonitrile solution. The mobile phases were used after the sonication. The loading volume, flow rate, gradient elution mode, and ion scan mode were referred to the method of Lu et al. [22]. 

The raw data files converted into the mzXML format by ProteoWizard software were processed by R package XCMS (version 3.2) for peak identification, peak extraction, peak alignment, and integration. The MS2 database and the Human Metabolome Database (HDMB) were used for metabolite annotation. Metabolite data were preprocessed (deviation value filtering, missing value filtering, missing value filling, and data normalization) and analyzed by MetaboAnalystR R package. Orthogonal partial least squares discriminant analysis (OPLS-DA) is based on partial least squares regression (PLS) and involves the reduction of high-dimensional data into a smaller number of orthogonal latent variables that maximize the separation between different classes or groups of data points, and it was used to select differential metabolites. In the OPLS-DA model, metabolites with a variable projection importance (VIP) greater than 1 were identified as differential metabolites. Kyoto Encyclopedia of Genes and Genomes (KEGG) compound IDs corresponding to differential metabolites were used for metabolic pathway enrichment analysis, which was conducted on MetOrigin (http://metorigin.met-bioinformatics.cn, accessed on 15 August 2022). The calculation of the *p*-value in this enrichment analysis was based on hypergeometric distribution. The enrichment ratio was calculated as the logarithm (base: 0.05) of the *p*-value.

### 2.9. Fecal Bacterial DNA Extraction and 16S rDNA Sequencing

A FastDNA Spin Kit for Feces (MP Biomedicals, Santa Ana, CA, USA) was used to extract microbial genomic DNA from feces, and the V3–V4 region of the extractions was amplified by primers 341F and 806R via a polymerase chain reaction. The amplified product was purified via electrophoresis on a 1.5% agarose gel, the gel was dissolved, and the purified product was collected. Samples whose DNA concentration did not meet the sequencing requirements were excluded. The Illumina MiSeq platform (Illumina, San Diego, CA, USA) was used for sequencing [23]. 

The Quantitative Insights into Microbial Ecology version 2 (QIIME2) platform was utilized to process the raw sequencing data for further analysis. Specifically, the operational taxonomic units (OTUs) were identified based on a 97% nucleotide identity threshold. The SILVA rRNA gene sequence database (v132) was used to classify the representative sequences of OTUs. Alpha diversity was evaluated by calculating the Shannon index and observed OTUs from rarefied OTUs. Bray−Curtis distances were utilized to estimate beta diversity, which was visualized by principal coordinate analysis (PCoA). In addition, the differential clustering of microbial communities was estimated by permutational multivariate analysis of variance (PERMANOVA) with vegan R package v2.5–6. In addition, linear discriminant analysis (LDA) effect size (LEfSe) was used to differentiate microbial biomarkers with a Wilcoxon-rank sum test (LDA score: >2.0, *p*-value: <0.05). Spearman’s correlation coefficients were used to construct a network, which was visualized using Gephi (v0.9.2).

### 2.10. Statistical Analysis 

Graphpad Prism version 8.4.3 was used to perform statistical analyses on body weight, serum biochemical indexes, quantification of immunohistochemistry, adipose tissue mass, and cytokines. Data are expressed as mean ± standard error of the mean (SEM). Significant differences between groups were evaluated using one-way analysis of variance (ANOVA), followed by Dunnett’s multiple comparison test against the HFD and NC groups. Statistical significance was set at a *p*-value of <0.05.

## 3. Results

### 3.1. C. butyricum Administration Reduces Diet-Induced Weight Gain and Hyperlipoidemia

**Figure 1:**
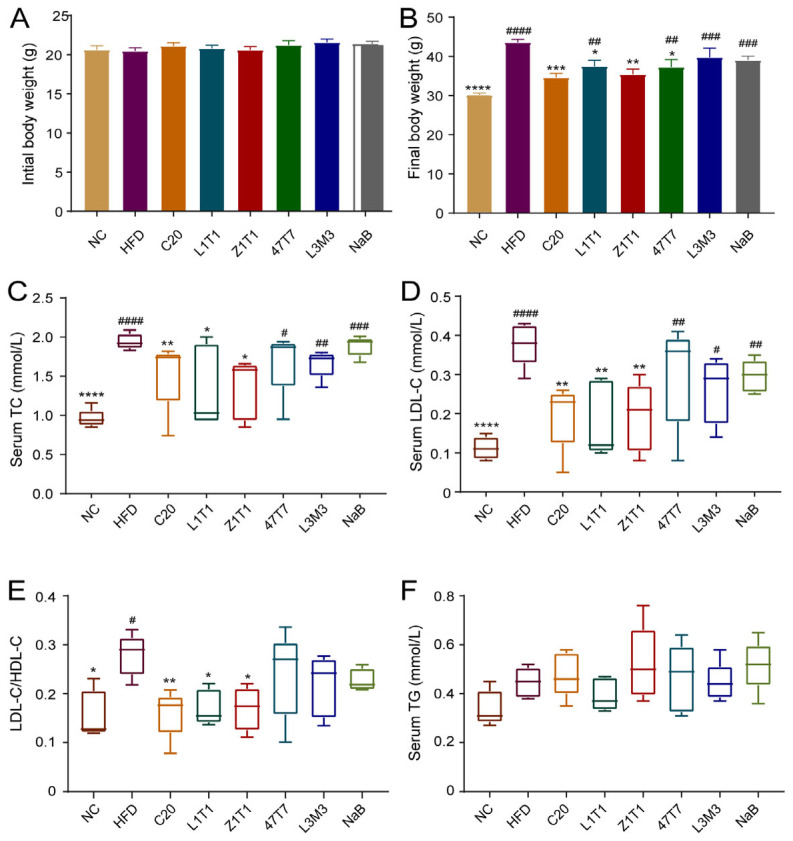
Effects of *C. butyricum* strains on body weight and dyslipidemia: (**A**) initial body weight of each group; (**B**) final body weights of mice on an HFD with or without *C. butyricum* administration and normal-chow (NC)-fed mice in the last week; (**C**) serum levels of TC; (**D**) serum levels of LDL-C; (**E**) ratio of LDL-C/HDL-C serum levels; (**F**) serum levels of TG. Data are shown as means with standard error of the mean (SEM); * (#), ** (##), *** (###), and **** (####) were used to indicate that the *p*-values were less than 0.05, 0.01, 0.001, and 0.0001, respectively, compared with the HFD group (versus the NC group). One-way ANOVA followed by Dunnett’s test for multiple comparison was used for significance analysis.

First, we evaluated the effect of *C. butyricum* (containing five *C. butyricum* isolates: C20, Z1T1, L1T1, 47T7, and L3M3) on body weight in mice fed with HFD for 12 weeks. Mice were orally given 300 mg/kg NaB to determine whether the probiotic benefits of *C. butyricum* can be replaced by butyrate. Mice fed with an HFD gained much more weight than mice fed with NCD. The administration of C20, L1T1, 47T7, and Z1T1 decreased body weight by 20%, 14%, 14%, and 19%, respectively (Figure 1B). Notably, C20 and Z1T1 almost nullified the effect of the HFD on body weight. The aforementioned comparisons were predicated on the notion that there was no variation in the initial body weight between the groups (Figure 1A). The serum concentrations of LDL-C, HDL-C, TC, and TG were determined to examine dyslipidemia, which is a primary characteristic of obesity. Because all strains did not significantly affect the level of HDL-C, we used the ratio of LDL-C to HDL-C to explore the influence of five strains on lipid transport in vivo. C20, Z1T1, and L1T1 alleviated HFD-induced hyperlipidemia, as these three strains reduced the serum levels of TC and LDL-C and the ratio of the LDL-C/HDL-C serum levels (Figure 1C–E). The TG levels in the serum did not differ across the groups (Figure 1F). In addition, NaB treatment failed to mimic the beneficial effects of *C. butyricum* on weight gain and did not improve dyslipidemia. Therefore, strains C20, Z1T1, and L1T1 can restrict body weight gain and prevent dyslipidemia, which could not be simply replaced by butyrate.

### 3.2. C. butyricum Administration Decreases Fat Mass and Alleviates the Adipose Inflammation

**Figure 2:**
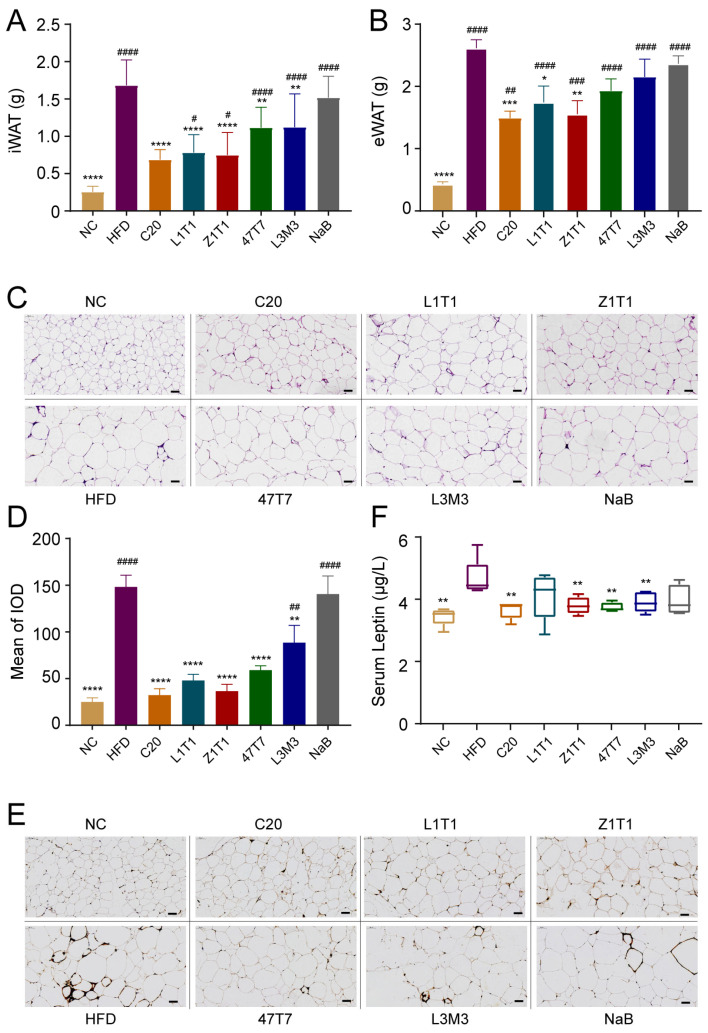
Effects of *C. butyricum* strains on adiposity and inflammation in adipose tissue: (**A**) mass of iWAT; (**B**) mass of eWAT; (**C**) representative iWAT hematoxylin–eosin (H&E) staining (scale bars: 50 μm; magnification: 200×); (**D**) quantification of the positive staining area; (**E**) representative images of adipose tissue F4/80 protein immunohistochemical staining. (**F**) Serum level of leptin. Data are shown as the means with SEM; * (#), ** (##), *** (###), and **** (####) were used to indicate that the *p*-values were less than 0.05, 0.01, 0.001, and 0.0001, respectively, compared with the HFD group (versus the NC group). One-way ANOVA followed by Dunnett’s test for multiple comparison was used for significance analysis.

As diet-induced obesity is associated with an excessive expansion of adipose tissue, the weight of adipose tissues and adipocyte size were determined to assess the effects of *C. butyricum* on adiposity. Epididymal white adipose tissue (eWAT) mass and inguinal white adipose tissue (iWAT) mass were substantially greater in the HFD group than in the NC group (Figure 2A,B), indicating that the HFD promotes body fat accumulation. All five *C. butyricum* strains showed decreased accumulation of iWAT; however, only C20, Z1T1, and L1T1 significantly inhibited the accumulation of eWAT. C20 and Z1T1 reversed HFD-induced adipocytes hypertrophy (Figure 2C), but NaB did not significantly inhibit the increase of adipose tissue weight and the hypertrophy of inguinal adipocytes (Figure 2A–C). Furthermore, all five *C. butyricum* strains, but not NaB, reduced the F4/80 positive staining area (represented by the mean of the integrated optical density (IOD)) in iWAT relative to that in the HFD group (Figure 2D,E). Consistently, under the influence of C20, Z1T1, 47T7, and L3M3, the pro-inflammatory adipokine leptin was also significantly lowered (Figure 2F). 

### 3.3. C. butyricum Administration Attenuates Fat Deposition and Inflammation in the Liver 

**Figure 3:**
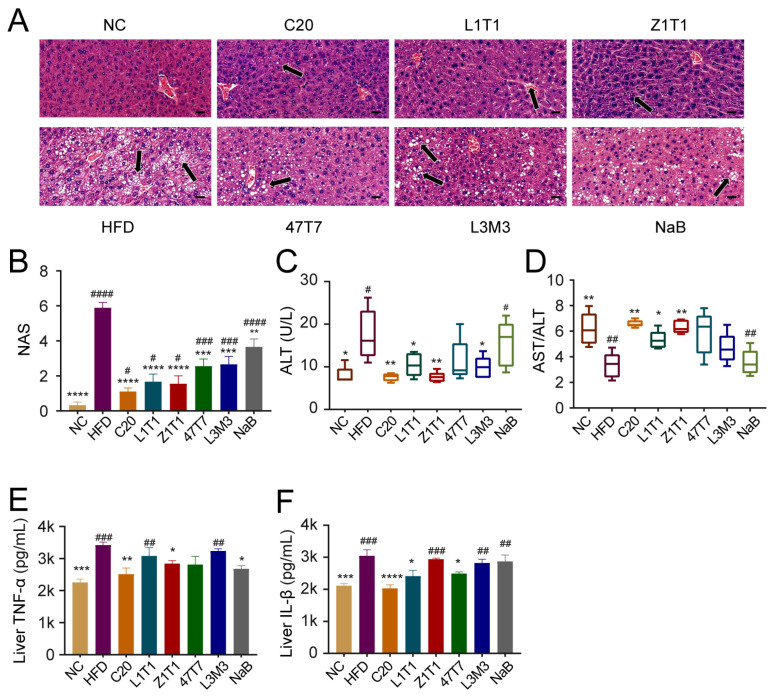
Effects of *C. butyricum* strains on hepatic steatosis and inflammation. (**A**) H&E staining of liver sections (scale bar: 20 μm; magnification: 400×). The black arrow shows the lipid droplet in liver; (**B**) NAFLD activity score (NAS) of each group; (**C**) serum level of ALT; (**D**) the ratio of AST/ALT serum levels; secretion of TNF-α (**E**) and IL-1β (**F**) in liver. Data are shown as means with SEM; * (#), ** (##), *** (###), and **** (####) were used to indicate that the *p*-values were less than 0.05, 0.01, 0.001, and 0.0001, respectively, compared with the HFD group (versus the NC group). One-way ANOVA followed by Dunnett’s test for multiple comparison was used for significance analysis.

Non-alcoholic fatty liver disease (NAFLD) is a prevalent obesity consequence associated with fat accumulation and inflammation in the adipose tissue. To investigate the effect of *C. butyricum* on the development of NAFLD, we first performed a histological study of the liver using hematoxylin–eosin (H&E) staining. The histological phenotype of NAFLD developed after long-term HFD intervention, which included liver steatosis and inflammatory infiltration (Figure 3A). Compared with the NC group, HFD-fed mice showed a higher NAFLD activity score (NAS) (Figure 3B). *C. butyricum* C20, Z1T1, and L1T1 prevented liver steatosis; however, NaB intervention was ineffective.

Supplementation with *C. butyricum* C20, Z1T1, and L1T1 rescued the clinical characteristics of the HFD group, which had a higher serum alanine aminotransferase (ALT) level and a lower aspartate aminotransferase (AST)/ALT serum level ratio (Figure 3C,D). The abnormal ALT levels and AST/ALT ratio in the blood of HFD-treated mice suggested liver inflammation; thus, we measured the expression of tumor necrosis factor (TNF)-α and interleukin (IL)-1β in the liver (Figure 3E,F). The TNF-α and IL-1β levels were elevated by the HFD. C20 could reverse the impact of the HFD on both cytokines; however, Z1T1, which effectively reversed diet-induced weight gain and hepatic steatosis, lowered only the level of TNF-α in the liver. L1T1 and 47T7 also helped reduce the levels of IL-1β. However, the TNF-α levels were also reduced by oral administration of NaB. This may be because butyrate initially reached the liver after being absorbed and most blood in the liver originated from the gut.

### 3.4. Administration of C. butyricum C20and Z1T1 Alters Purine and Tryptophan Metabolism in Stool 

**Figure 4:**
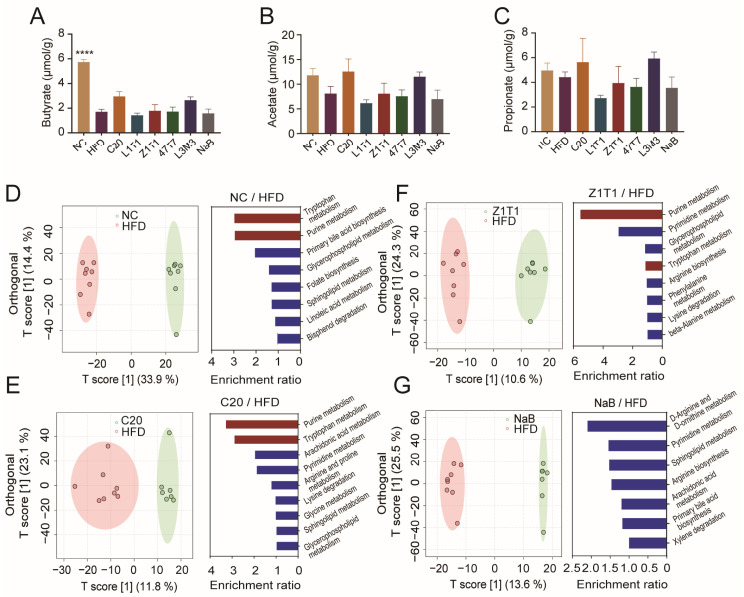
Effect of *C. butyricum* strains on intestinal metabolites: intestinal levels of butyrate (**A**), acetate (**B**), and propionate (**C**); OPLS-DA and enrichment analysis between the NC (**D**), C20 (**E**), Z1T1 (**F**), NaB (**G**), and HFD groups. Data are shown as means with SEM; **** was used to indicate that the *p*-values was less than 0.0001, compared with the HFD group. One-way ANOVA followed by Dunnett’s test for multiple comparison was used for significance analysis.

Although daily treatment with NaB did not effectively prevent diet-induced obesity, this did not prove the ineffectiveness of bacteria-produced SCFAs. Exogenous supplementation and endogenous synthesis may result in distinct SCFA concentrations in the intestine. To investigate whether oral administration of *C. butyricum* altered the compositions and the concentrations of intestinal SCFAs, we examined the concentration of butyrate, acetate, and propionate in the cecal contents using GC-MS. However, none of the *C. butyricum* and NaB treatments restored the concentrations of these three SCFAs in the cecum (Figure 4A–C), indicating that the inhibition of body weight gain by *C. butyricum* C20 and Z1T1 was not achieved by increasing the intestine luminal concentrations of SCFAs. Based on the fact that gut derivatives constitute the primary link between the microbiota and the host, we further performed an untargeted metabolomic analysis of stool using LC-MS. OPLS-DA revealed changes in the composition of intestinal material across the NC, C20, Z1T1, NaB, and HFD groups (Figure 4D–G). In addition, the enrichment analysis of the differential metabolites between the NC and HFD groups revealed that tryptophan and purine metabolism were the two most enriched pathways (Figure 4D). The administration of C20 and Z1T1, the two most efficacious strains against diet-induced obesity, concurrently enhanced the levels of purine and tryptophan metabolites (Figure 4E,F). This may explain why these two strains were more successful than NaB in suppressing obesity, as NaB did not affect these two metabolic pathways (Figure 4G).

### 3.5. Effects of C. butyricum on the Gut Microbial Composition of HFD-Treated Mice

**Figure 5:**
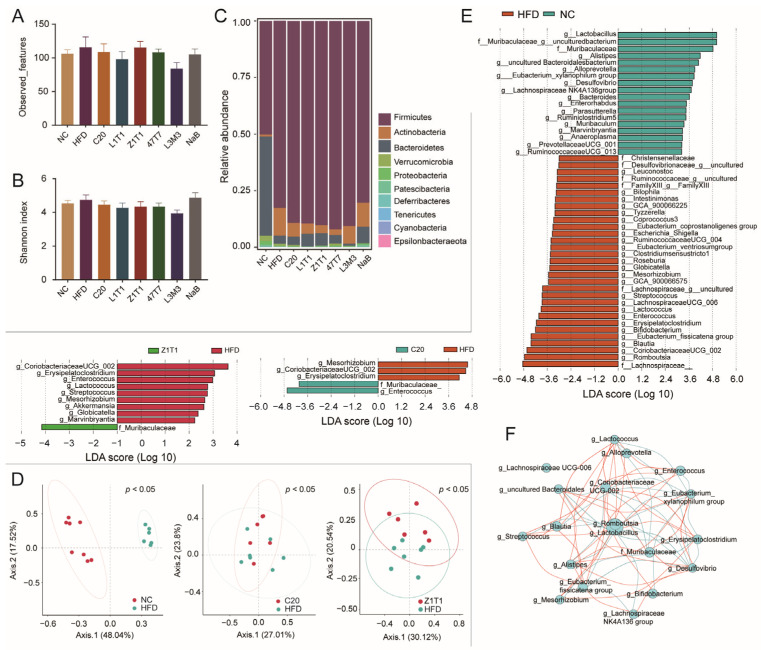
Impact of *C. butyricum* on the gut microbiota: (**A**) observed_features; (**B**) Shannon index; (**C**) microbial composition of each group at the phylum level; (**D**) PCoA results (left: HFD/NC, *p* = 0.001; middle: HFD/C20, *p* = 0.031; right: HFD/Z1T1, *p* = 0.012;); (**E**) LEfSe between the NC, C20, Z1T1, and HFD groups (LDA score: >2; *p*-value: <0.05); (**F**) correlation analysis results among differential genera. Differences were analyzed via one-way ANOVA followed by Dunnett’s multiple comparison test.

The microbiota can be influenced by diet and is linked to the development of obesity. To investigate the effect of *C. butyricum* on the composition of the gut microbiota, we performed 16S rDNA amplicon sequencing of stool samples. According to the observed features and Shannon index, we found that α-diversity did not differ between the groups (Figure 5A,B). We further explored whether there were differences in microbial composition at the phylum level (Figure 5C) and β-diversity (Figure 5D). Principal coordinate analysis (PCoA) revealed a significant difference in β-diversity among the NC, C20, Z1T1, and HFD groups. At the phylum level, the HFD enriched not only *Firmicutes*, but also *Actinobacteria*. Although five *C. butyricum* strains failed to decrease *Firmicutes*, strains C20, Z1T1, 47T7, and L1T1 reduced the proportion of *Actinobacteria*. 

We investigated a linear discriminant analysis effect size (LEfSe) to identify the distinct bacteria at different taxonomic levels among the C20, Z1T1, HFD, and NC groups. Compared to the NCD, the HFD increased the abundance of 31 genera that can be classified into 15 families, with approximately half of those genera belonging to the families *Ruminococcaceae* and *Lachnospiraceae* (Figure 5E). The administration of C20 and Z1T1 restrained the growth of *g_Mesorhizobium*, *g_Coriobacteriaceae_UCG_002*, and *g_Erysipelatoclostridium*, while increasing the growth of the genera from *f_Muribaculaceae*, which were also abundant in the NC group (Figure 5E). In addition, Z1T1 reduced the abundance of *g_Enterococcus*, *g_Lactococcus*, *g_Streptococcus*, *g_Globicatella*, and *g_Marvinbryantia*. This suggests that *C. butyricum* has strain-specific effects on the microbiota and ultimately leads to differences in the ability of the strains to resist diet-induced obesity. Correlation analysis revealed that *f_Muribaculaceae* was negatively associated with *g_Alistipes*, *g_Erysipelatoclostridium*, and *g_CoriobacteriaceaeUCG_002* (Figure 5F).

## 4. Discussion

Microbial preparations have been intensively investigated for decades as a primary strategy for combating obesity. The anti-obesity effects of probiotic *Lactobacillus* and *Bifidobacterium* strains have been confirmed not only in animal models, but also in clinical trials [24]. The abundance of *C. butyricum* was found to be decreased in obese individuals, and it has been shown that the orally administration of *C. butyricum* can alleviate the phenotype of obesity [25]. This butyrate-producing symbiont can stimulate the secretion of gastrointestinal hormones and alter the function of the parenteral organ [26]. It can also directly regulate the functioning of adipose tissue and other extraintestinal tissues by releasing its metabolites or gut-derived substances into the circulatory blood [27]. Our findings indicated that *C. butyricum* can restrain the development of obesity, which is consistent with prior studies, and two effective strains (C20 and Z1T1) have been selected in this study. These two effective strains can not only attenuate weight gain, but also ameliorate dyslipidemia and reduce adipose tissue mass. 

Obesity and metabolic illnesses are characterized by chronic inflammation. Macrophages are innate immune cells that are essential for maintaining homeostasis, and macrophage infiltration into adipose tissue is usually present in obese individuals [28]. Our preliminary investigation demonstrated that five *C. butyricum* strains could reduce the HFD-induced macrophage infiltration in adipose tissue. As the recruitment of monocytes and macrophages is associated with adipose tissue dysfunction [29], we suggested that *C. butyricum* could alleviate obesity by targeting macrophages in adipose tissue, and its effect on the function of adipose tissue requires additional study. 

Obesity-related adipose-tissue inflammation contributes to the onset of subsequent metabolic diseases, one of which is NAFLD. As the “first hit” in the pathogenesis of NAFLD, hepatic steatosis is associated with inflammation-induced lipolysis in adipose tissue [30]. Since adipose-tissue inflammation was suppressed, we speculated that hepatic steatosis could also be improved by *C. butyricum*. The H&E staining of liver sections revealed that intrahepatic fat accumulation was mitigated to varying degrees by five strains, with strains C20, Z1T1, and L1T1 having the most significant effects. Inflammation is the “second hit” in the pathogenesis of the NAFLD, leading to the transition from hepatic steatosis to nonalcoholic steatohepatitis (NASH) [31]. TNF-α and IL-1β, two NASH-related cytokines [32], were measured to assess the effect of *C. butyricum* on liver inflammation. Treatment with C20 significantly lowered the expression of both cytokines simultaneously. Z1T1 decreased the expression of TNF-α, whereas L1T1 and 47T7 reduced the intrahepatic levels of IL-1β. Serum ALT levels were also measured. C20, Z1T1, and L1T1 significantly reduced the ALT levels, indicating that parenchymal damage to the liver was ameliorated. The therapeutic effect of *C. butyricum* on NAFLD has been demonstrated in both animal models and clinical trials [33]. Although *C. butyricum* has been associated with obesity and its associated morbidities, its regulatory influence on the gut microbiota and the gut derivatives for the treatment of illnesses remains unknown. 

The gut derivatives are associated with the etiology of metabolic diseases, because they can serve as signaling molecules to guide the physiological processes of the host, and they can also enter the systemic circulation to act directly on peripheral tissues. Although certain study has pointed out that butyrate can promote adipose tissue thermogenesis by activating lysine specific demethylase 1 (LSD1) [34], others demonstrate that butyrate enhances lipogenesis in the liver and adipose tissue [35]. In this study, we found that the beneficial effects of *C. butyricum* administration could not be replaced by NaB supplementation and the level of butyrate in the intestine did not change significantly with the intervention of *C. butyricum*. Therefore, the anti-obesity effects of *C. butyricum* might not be achieved by producing butyrate, and this should be verified by gene editing in the future.

Because the level of butyrate in the intestine was not significantly increased, we further explored the effect of *C. butyricum* on the composition of the gut derivatives via LC-MS. Compared to the HFD group, the intervention with *C. butyricum* C20 and Z1T1 both altered the metabolism of tryptophan and purines in the gut, but NaB failed to change these two metabolic pathways. Thus, we hypothesized that purine and tryptophan metabolites are associated with the anti-obesity effects of these two strains. The microbial metabolism of tryptophan mainly produces indole derivatives, which are related to the onset and development of obesity. Certain indole derivatives can prevent the development of obesity [10,36], and the capacity of the gut bacteria to convert tryptophan to indole also increases after gastric bypass surgery [37]. Mechanistically, indole inhibits the expression of miRNA181 in white fat and promotes thermogenesis in adipose tissue [38]. In addition to indole, indole acetic acid and indole propionic acid also could resist the occurrence of diet-induced obesity [39]. Considering that *Clostridium* genus is one of the main genera to synthesize indole derivatives [40], the colonization rates and capacities of these five strains to produce indole derivatives should be verified in vitro and in vivo. Adenosine and inosine are the purine metabolites that are generated, when CD39 degrades extracellular adenosine triphosphate (ATP) [41], which can enhance the thermogenic capacity of adipose tissue by increasing intracellular cyclic adenosine monophosphate (cAMP) levels [42] or indirectly reduce obesity by modulating the host immunity [43]. Studies have shown that the expression of CD39 is related to AhR activation. Therefore, indole derivatives, ligands of AhR, not only directly participate in the regulation of the host’s metabolism and the immune system, but also might play an indirect role by promoting the production of adenosine and its metabolite inosine [44]. Further research is required to determine whether *C. butyricum* C20 and Z1T1 ameliorate obesity by enhancing the crosstalk between the purinergic and AhR signaling pathways.

The gut microbiota of obese individuals is distinct from that of lean individuals, and it is associated with obesity. After colonizing the gut microbiota in germ-free mice, lipid absorption in the intestine was enhanced under both a high-fat and low-fat diets [45]. In addition, transplanting the fecal microbiota of the obese individual in twins into mice makes them more susceptible to obesity [46]. Greater abundances of *Enterobacteriaceae*, *Coprococcus*, and *Eubacterium* have been reported in several studies [47,48], which was also observed in our study. Consistent with the findings of Ottosson et al., *Blautia* was more prevalent in the HFD group in our study [49]. However, there may be interspecific differences in the effect of *Blautia* on body weight management, as some evidence suggests that the abundance of *Blautia* is inversely associated with visceral fat accumulation and that *Blautia wexlerae* supplementation can alleviate obesity-related metabolic syndromes [50,51]. This reminded us that metagenomic sequencing, a more precise technique, should be utilized to investigate the effect of *C.butyricum* on the microbiota. Two effective strains, C20 and Z1T1, restored the abundance of *f_ Muribaculaceae*, which was drastically decreased by the HFD and was inversely linked with the development of obesity [52,53]. Considering the association between *f_Muribaculaceae* and obesity, the greater abundance of *f_Muribaculaceae* in the NC, C20, and Z1T1 groups suggested that *f_Muribaculaceae* may be a biomarker of obesity. The molecular mechanism by which *f_Muribaculaceae* relieves obesity and its interaction with *C. butyricum* warrants further research.

## 5. Conclusions

Obesity accelerates human aging and causes various metabolic diseases. The gut microbiota has been regarded as the main target of obesity treatment, and microbiome therapy is one of the practical means. Thus, screening for more microbial agents with anti-obesity effects is a hotspot in this field. In this study, we discovered that treatment with two *C. butyricum* strains could reduce the harmful effects of the HFD and alleviated obesity-related syndromes. Furthermore, through an in-depth exploration of the mechanism of effective strains, we found that the anti-obesity effect of *C. butyricum* was not mediated by butyrate, but probably by altering the purine and tryptophan metabolism in the gut or regulating the structure of the gut microbiota. Our data revealed that *C. butyricum* can resist obesity and will assist further studies on the mechanism by which *C. butyricum* inhibits diet-induced obesity. In addition to studying its beneficial effects, the evaluation of its safety should be systematically performed in the future, including antibiotic resistance and reproductive and developmental toxicity. 

## Data Availability

The raw 16S rDNA data in this study can be found by project accession no. PRJNA898110 at https://www.ncbi.nlm.nih.gov/ (accessed on 5 November 2022).
